# Lung Ultrasound Imaging Dataset for Accurate Detection and Localization of LUS Vertical Artifact

**DOI:** 10.1038/s41597-025-05854-4

**Published:** 2025-10-16

**Authors:** Nixson Okila, Andrew Katumba, Joyce Nakatumba-Nabende, Sudi Murindanyi, Cosmas Mwikirize, Jonathan Serugunda, Samuel Bugeza, Anthony Oriekot, Juliet Bossa, Eva Nabawanuka

**Affiliations:** 1 Department of Computer Science, Makerere University, Kampala, Uganda; 2 Department of Electrical and Computer Engineering, Makerere University, Kampala, Uganda; 3 Emergent AI, Kampala, Uganda; 4 Department of Radiology, Makerere University Hospital, Makerere University, Kampala, Uganda; 5 Mulago Specialized Women and Neonatal Hospital, Kampala, Uganda; 6 Mulago National Referral Hospital, Kampala, Uganda

**Keywords:** Biomarkers, Electrical and electronic engineering

## Abstract

Lung ultrasound (LUS) vertical artifacts are critical sonographic markers commonly used in evaluating pulmonary conditions such as pulmonary edema, interstitial lung disease, pneumonia, and COVID-19. Accurate detection and localization of these artifacts are vital for informed clinical decision-making. However, interpreting LUS images remains highly operator-dependent, leading to variability in diagnosis. While deep learning (DL) models offer promising potential to automate LUS interpretation, their development is limited by the scarcity of annotated datasets specifically focused on vertical artifacts. This study introduces a curated dataset of 401 high-resolution LUS images, each annotated with polygonal bounding boxes to indicate vertical artifact locations. The images were collected from 152 patients with pulmonary conditions at Mulago and Kiruddu National Referral Hospitals in Uganda. This dataset serves as a valuable resource for training and evaluating DL models designed to accurately detect and localize LUS vertical artifacts, contributing to the advancement of AI-driven diagnostic tools for early detection and monitoring of respiratory diseases.

## Background & Summary

Lung ultrasound (LUS) has gained prominence as a valuable imaging modality in pulmonary and critical care medicine, owing to its portability, real-time imaging capabilities, and non-ionizing nature^1^. Among the sonographic features identifiable in LUS, vertical artifacts, arising from resonance phenomena when ultrasound waves interact with fluid-filled alveoli or thickened interlobular septa in areas of increased pulmonary density, are particularly significant for evaluating pulmonary congestion^2,3^. These artifacts serve a crucial role in the assessment of a broad spectrum of respiratory and systemic conditions, including pulmonary edema, decompensated heart failure, chronic kidney disease, pneumonia, interstitial lung disease, and COVID-19^4^. Despite the diagnostic utility of LUS, the modality inherently lacks anatomical imaging of the lung parenchyma^1^. Consequently, clinical interpretation relies on the accurate recognition and analysis of artifact patterns, a process that is susceptible to considerable inter- and intra-observer variability, as demonstrated in recent studies^5–7^. In a study involving LUS of COVID-19 patients, Lerchbaumer *et al*.^5^ evaluated agreement among ten observers who each rated 100 cine loops four times. The results indicated fair to moderate inter-observer agreement (Fleiss’ κ ≈ 0.27 for intermediate scores and ~0.59 for subpleural consolidations) and moderate to substantial intra-observer agreement (κ up to ~0.71–0.79 for distinct features). Similarly, Fatima *et al*.^6^ evaluated interrater agreement among six LUS experts for both video-level and and prognostic-level scoring. At the video level, a kappa value of 0.404 was reported for overall scores, while at the prognostic level, the kappa value was 0.505, both reflecting moderate agreement among the experts. The findings underscore the inherent subjectivity associated with expert interpretation of LUS. In a related study^7^, interrater agreement was assessed between three LUS experts and AI-based solution at the video level. the mean kappa value for overall scores among the experts was 0.61, also indicating moderate agreement and further highlighting the subjective nature of human analysis.

To mitigate the challenges of LUS interpretation, recent studies^8–12^ have focused on leveraging deep learning (DL) techniques to automate LUS image analysis, with a particular focus on the detection and localization of LUS vertical artifacts. While these methods show considerable promise, their effectiveness remains constrained by the scarcity of annotated datasets designed explicitly for this task. In particular, the lack of datasets with detailed polygonal bounding box (PBB) annotations has hindered the development of models capable of accurately capturing the morphology and spatial distribution of LUS vertical artifacts. This limitation often results in models that exhibit suboptimal performance, such as imprecise artifact delineation and inclusion of extraneous background regions, thereby reducing diagnostic accuracy and increasing computational burden.

To address this gap, we introduce the LUS-BALD (Lung Ultrasound – LUS Vertical Artifact Localization and Detection) dataset, the first publicly available resource containing LUS images with corresponding PBB annotations for LUS vertical artifacts. The dataset comprises 401 images, partitioned at the patient level into training, validation, and test subsets, with annotations provided for the training and validation sets. The LUS-BALD dataset is intended to facilitate the development and benchmarking of deep learning algorithms for precise LUS vertical detection and localization, and it holds potential for practical applications such as AI-assisted educational platforms for training healthcare providers in LUS interpretation.

## Methods

**Fig. 1:**
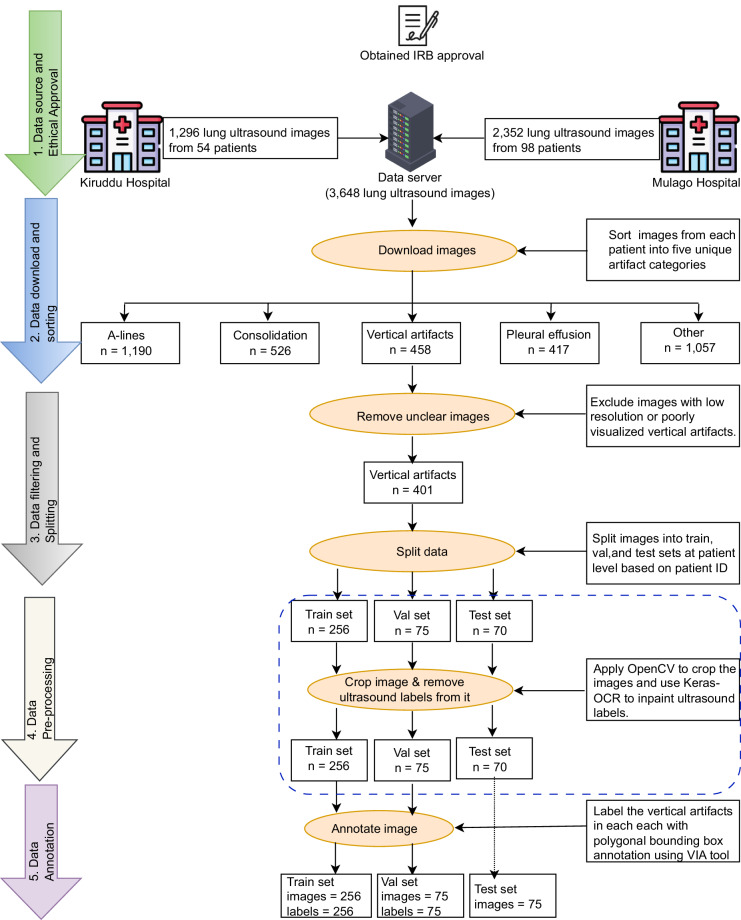
Workflow for LUS-BALD dataset collection and curation. The process begins with ethical approval and data acquisition, followed by sequential stages of data filtering, image preprocessing, and detailed annotation.

The methodological workflow for the systematic collection and curation of the LUS-BALD dataset is illustrated in Fig. 1.

### Participants and ethical requirements

A total of 152 participants (113 males and 39 females; mean age: 40.91 ± 17.03 years) were recruited for a clinical study conducted at Mulago and Kiruddu National Referral Hospitals in Kampala, Uganda. The cohort comprised individuals with suspected COVID-19 infection, as well as patients presenting with other pulmonary pathologies, including cases of non-COVID-19 pneumonia. Ethical approval for the study was obtained from the Mulago Hospital Research and Ethics Committee (MHREC; Approval No. MHREC 2021-45, dated 24 August 2021) and the Uganda National Council for Science and Technology (UNCST; Approval No. SIR74ES, dated 22 October 2021).

Informed written consent was obtained from all participants prior to enrolment. The consent process included a comprehensive explanation of the study objectives, data collection procedures, and participant rights. Participants were informed of their right to withdraw from the study at any time without consequence. Consent also included permission for the use of anonymized data for research purposes, with explicit assurance that personal identifiers would remain confidential.

### Data collection

The LUS imaging was conducted by three senior radiologists, each with over ten years of clinical experience in LUS. Two handheld curvilinear ultrasound probes were used: the Clarius C3 (Clarius Mobile Health Corp., Burnaby, BC, Canada) and the Philips Lumify C5-2 (Philips Ultrasound, Bothell, WA, USA). Acquisition settings were harmonized across devices: Clarius C3 operated at 2–6 MHz with a mechanical index (MI) ≤ 0.68, and Philips Lumify C5-2 at 2–5 MHz with MI approximately 0.7. The focal depth was positioned at the pleural line (~3 cm), with a frame rate of 20 Hz, gain set to 53, and power output at −0.3 dB. Although a depth of 16 cm was uniformly applied in our acquisitions to capture the complete ultrasound field, the key LUS features (pleural line and vertical artifacts) were identifiable within the pleural line depth of 2–4 cm. This 2–4 cm depth range is the consistent with previous reported findings^13–15^.

During the LUS scanning, radiologists divided each patient’s thorax into 12 regions—six on the left (upper posterior, lower posterior, upper anterior, lower anterior, upper lateral, lower lateral) and six on the right (upper posterior, lower posterior, upper anterior, lower anterior, upper lateral, lower lateral). Both longitudinal and transverse scans were performed in each region, generating a total of 24 image frames per patient, each stored in a unique folder labelled with the corresponding patient ID and uploaded to the data server.

Segmenting the thorax into these 12 distinct zones enabled radiologists to systematically evaluate different lung areas and detect localized abnormalities that could vary across regions^16^. Specifically, dividing the thorax into upper and lower, anterior, posterior, and lateral sections on both sides allowed for a more precise assessment of the distribution and severity of lung involvement^17^. Additionally, scanning both longitudinally and transversely within each region provided multiple perspectives, enhancing the detection of hidden pathologies that might otherwise be overlooked with a single scan orientation^16^.

### Data filtering and splitting

We obtained 458 images with LUS vertical artifacts from the data server and, in consultation with radiologists, excluded 57 low-resolution images and those with unclear artifacts. To prepare the dataset for model training, we partitioned the images into train, val, and test splits at the patient level, ensuring that images from the same patient appeared in only one partition.

### Data pre-processing

The LUS images included overlaid measurement scales and text labels. To eliminate these artifacts, a two-step pre-processing approach was employed: (i) image cropping to remove regions containing measurement scales, and (ii) inpainting techniques to remove overlaid text labels.

**Fig. 2:**
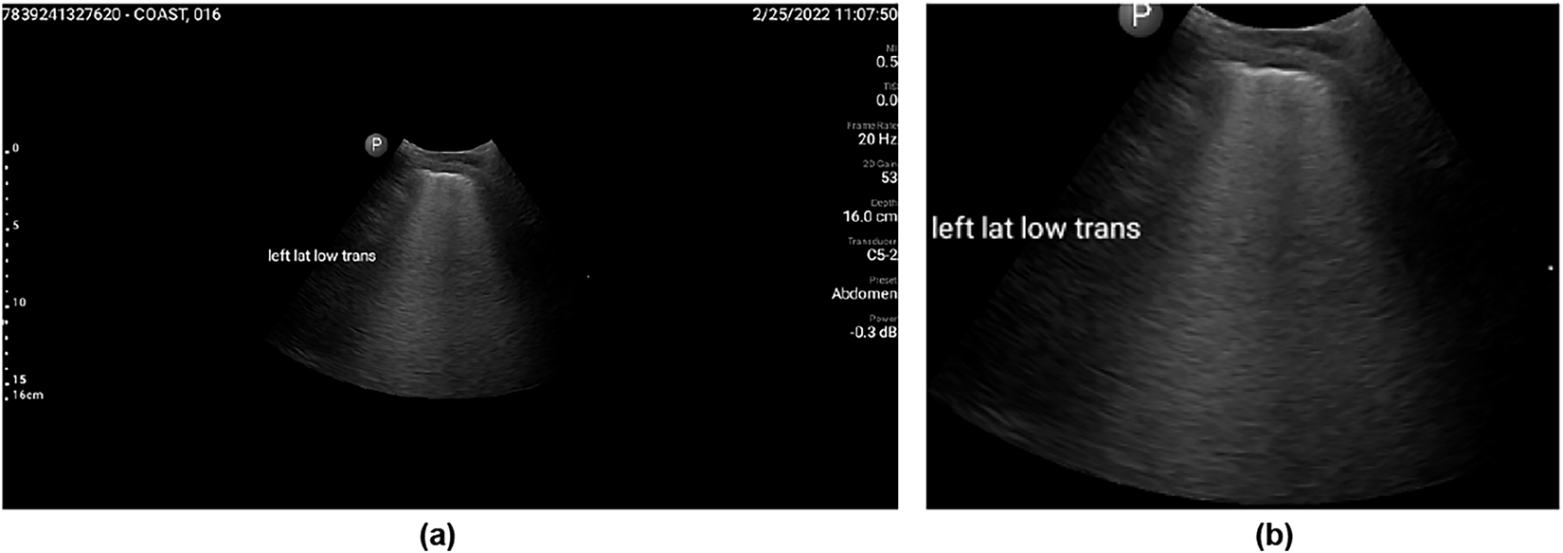
Preprocessing step for removal of overlaid measurement scales and text labels from lung ultrasound images. (**a**) Input image with overlaid measurement scale and text labels. (**b**) Cropped image containing only the text labels, with the measurement scale removed.

The image cropping procedure commenced by loading the input image using OpenCV and converting it to grayscale to reduce computational complexity while preserving essential structural information. Subsequently, Otsu’s thresholding method was applied to binarize the image by automatically selecting an optimal threshold to differentiate foreground structures from the background. To enhance segmentation quality, morphological operations were performed. Specifically, morphological closing with a small rectangular kernel was employed to fill small gaps within detected structures, ensuring their continuity. This was followed by dilation to strengthen object boundaries and facilitate more accurate contour detection. Upon completion of pre-processing, contours were identified using OpenCV’s contour detection algorithm. Each contour was evaluated based on its area, with the largest contour selected as the primary region of interest. A bounding box was then constructed around this region to delineate its spatial extent. The final step involved cropping the image using the computed bounding box, thereby isolating the principal structure from the background, as illustrated in Fig. 2.

**Fig. 3:**
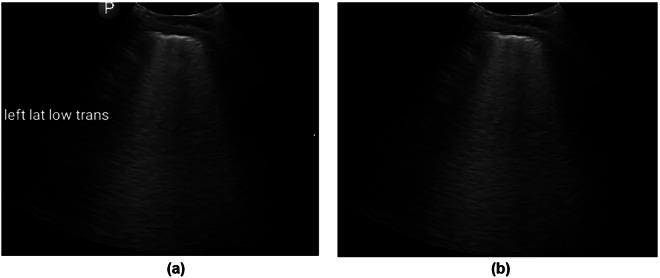
Text label removal from lung ultrasound images. (**a**) Cropped image with overlaid text labels. (**b**) Processed image after text detection, masking, and inpainting, resulting in an image free of text labels.

To eliminate residual text labels from the ultrasound images, the Keras-OCR library was employed for text detection and recognition. The library identifies textual elements by extracting bounding box coordinates around detected text instances, each represented as a quadrilateral defined by four corner points. Following text detection, a binary mask was generated to localize the text regions. For each quadrilateral, two midpoints were calculated along its vertical edges, serving as anchor points for drawing a thick line across the text region. The line thickness was adaptively determined based on the Euclidean distance between adjacent text coordinates to ensure complete coverage of the text. The resulting mask was then processed using OpenCV’s inpainting algorithm, which reconstructed the masked regions by interpolating pixel information from the surrounding areas. This procedure effectively removed text overlays while preserving the structural integrity of the underlying anatomical features. The processed image, free from textual annotations, is shown in Fig. 3(b).

### Data annotation

**Fig. 4:**
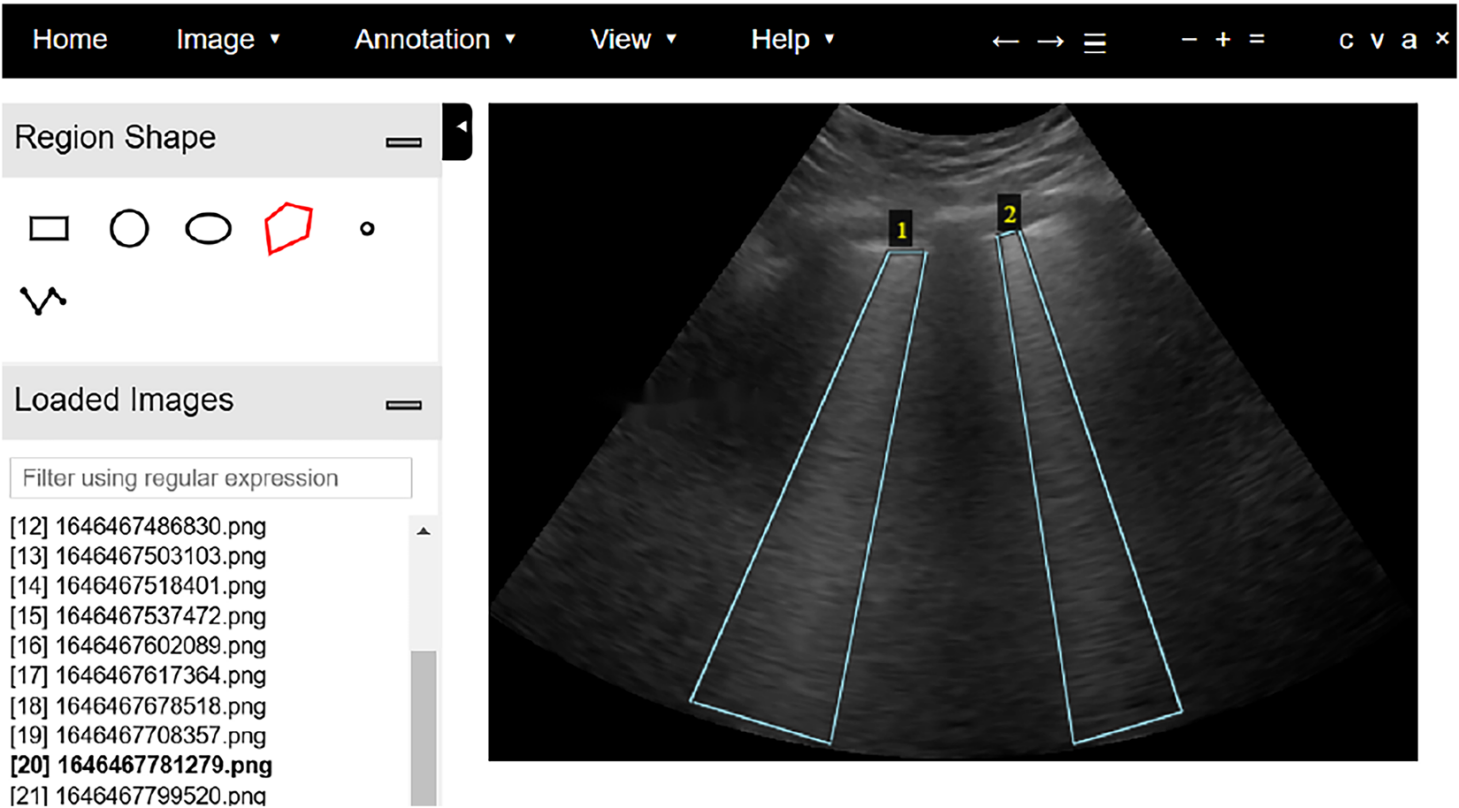
Annotation using the VGG Image Annotator. The figure illustrates two instances of annotated lung ultrasound vertical artifacts.

The annotation of LUS vertical artifacts was performed by manually delineating bounding boxes around the regions of interest in the images. An open-source tool, the VGG Image Annotator (VIA)^18^, was utilized for this task. The VIA tool supports multiple annotation formats, including polygonal bounding boxes, which were selected for their flexibility in accurately capturing the irregular shapes of LUS vertical artifacts. The annotation process involved launching the VIA interface, uploading the collected images, and collaboratively annotating each image in the training and validation sets in consultation with experienced radiologists. Polygonal bounding boxes were drawn around each identified LUS vertical artifact, and the corresponding annotations were systematically saved. Figure 4 presents an example of the annotation process in VIA, illustrating two instances of LUS vertical artifacts annotated with polygonal bounding boxes.

## Data Records

**Fig. 5:**
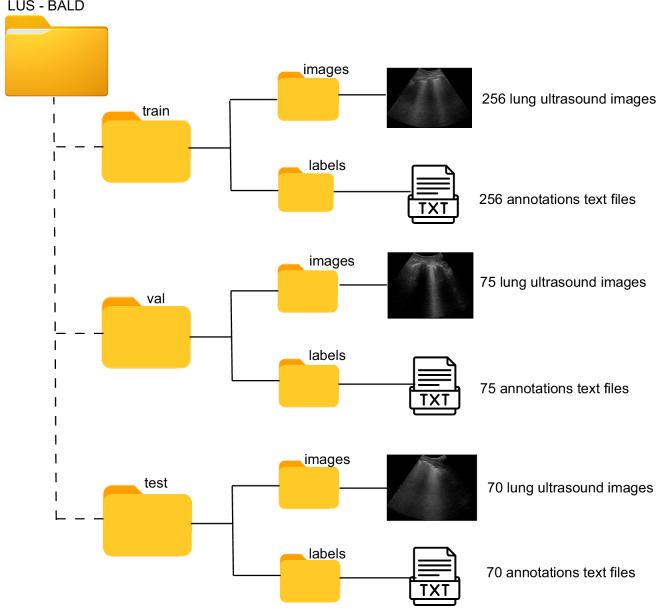
Directory structure of the LUS-BALD dataset. The figure presents the hierarchical organization of files and folders within the LUS-BALD dataset.

The dataset is available at Mendeley Data^19^. The root directory of the repository, named LUS-BALD, is organized into three principal subdirectories: train, val, and test, following a conventional partitioning strategy employed in supervised learning paradigms as shown in Fig. 5.

The train directory comprises two subfolders:

- images: Contains 256 lung ultrasound (LUS) image files in .png and .jpg formats.
- labels: Consists of 256 annotation files in YOLO (You Only Look Once) format, adapted to include four-vertex polygonal bounding boxes for the precise localization of LUS vertical artifacts. Each annotation file is paired with a corresponding image file.

The val directory mirrors the structure of the training set, containing 75 LUS image files and their corresponding annotation files, organized under images and labels subfolders, respectively.

The test directory contains a single images subfolder, comprising 70 LUS image files in .png and .jpg formats. Annotation files are not included for this subset, reflecting its intended use for model evaluation in a blind testing scenario.

## Technical Validation

To ensure the reliability and utility of the dataset, multiple validation steps were incorporated throughout data acquisition, annotation, and quality control. Senior radiologists acquired lung ultrasound images following a standardized 12-zone scanning protocol. Imaging was performed using a convex probe in both longitudinal and transverse orientations, with each image systematically recorded and labelled according to the corresponding lung zone.

Following the acquisition, the images were reviewed and curated in collaboration with radiologists to ensure quality and clinical relevance. Images exhibiting motion artifacts, poor pleural line visibility, or incomplete anatomical views were excluded. Ultimately, a curated subset of 401 high-resolution LUS images containing LUS vertical artfact artifacts was selected based on imaging clarity and diagnostic significance.

The annotation of LUS vertical artifacts was conducted by the authors in collaboration with radiologists, utilizing a custom labelling interface to apply polygonal bounding boxes that precisely delineated the shape and orientation of each artifact. To ensure annotation consistency and reliability, a two-stage validation process was employed. A second expert radiologist independently reviewed the initial annotations, and any discrepancies were resolved through consensus adjudication. This rigorous validation approach minimized inter-observer variability and ensured accurate and reproducible representation of LUS vertical artifact features within the dataset.

A key limitation of the dataset lies in its reliance on high-resolution LUS images curated primarily for artifact clarity, which constitutes on idealized representation of lung ultrasound data. In clinical settings, the identification of vertical artifacts generally requires evaluation of full cine loops that often contain motion artifacts and considerable variability in image quality. Moreover, only a single representative frame was acquired per scanned region, rather than full video sequences. While this approach was prectical for dataset curation, it fails to capture the temporal dynamics inherent to LUS and my introduce operator-dependent bisas in frame selection. These constraints may limit the dataset's capacity to fully represent the variability and complexity of real-world LUS interpretation. Future dataset development should therefore emphasize acquisition of complete video sequences to mitigate selection bias and preserve the dynamic characteristics of LUS.

## Data Availability

The code for preprocessing LUS-BALD images is available at https://github.com/Marconi-Lab/lus-bline-artifact-prep/.
